# Thickness of the aqueous boundary layer in stirred microtitre plate permeability assays (PAMPA and Caco-2), based on the Levich equation^ǂ^

**DOI:** 10.5599/admet.1568

**Published:** 2022-12-06

**Authors:** Alex Avdeef

**Affiliations:** in-ADME Research, 1732 First Avenue #102, New York, NY 10128 USA. E-mail: alex@in-ADME.com; Tel.: +1-646-678-5713

In permeability assays using microtitre plates, either based on cellular models (*e.g*., Caco-2, MDCK) or PAMPA (parallel artificial membrane permeability assay) [1-3], the thickness of the aqueous boundary layer (ABL) has been approximated by:


(1)

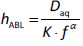



where *D*_aq_ is the diffusivity, *f* is the stirring frequency (RPM), and *K* and α are fitted constants [4-9].

Based on testosterone Caco-2 measurements, Karlsson and Artursson [5] reported α = 1 and implied *K* = 0.57 x 10^-6^ cm/s. Adson *et al.* [6] reported *K* = 4.1 x 10^-6^ cm/s and α = 0.8, also for testosterone. Orbital shakers were used to agitate the microtitre plates during the assay, as is the common practice in cellular assays. Both groups noted that the *K* parameter is a function of aqueous diffusivity, kinematic viscosity, and geometrical factors. Adson *et al.* [6] pondered on the α factor being greater than the theoretically expected value of 0.5 and reasoned that the asymmetric hydrodynamic conditions of the Transwell plates may have led to the elevated values.

In a PAMPA study of 53 ionizable molecules, Avdeef *et al.* [9] determined the ABL permeability, *P*_ABL_, using the p*K*_a_^flux^ method at four different stirring speeds (49, 118, 186, 622 RPM). Efficient individual-well magnetic stirring (using the Gut-Box device) was used in their study. Since *P*_ABL_ = *D*_aq_ / *h*_ABL_, the constants *K* and α can be determined for each molecule by linear regression based on log *P*_ABL_ = log *K* + α log *f*. The p*K*_a_^flux^ method uniquely made such an analysis possible. The least-squares refined parameters (based on several molecules) were reported as *K* = 23.1 x 10^-6^ cm/s and α = 0.709. The implicit assumption in the analysis was that for a given rate of stirring, there is a unique ABL thickness for all molecules.

In the above three studies, different values of α were reported, all greater than the theoretical value of 0.5 expected from the solution to the convective diffusion model partial differential equation, based on the rotating disk geometry, according to Levich [10]. In the theoretical model, the thickness of the ABL may be calculated from:


(2)

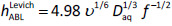



where *ν* is the kinematic viscosity (cm^2^/s). If the Levich equation were applicable to microtitre plate permeability assay geometries, then Eq. (2) suggests that *K* = 0.201 *ν*^- 1/6^ *D*_aq_^2/3^, provided α were 0.5 in Eq. (1). Hence, each molecule in the permeability assay would be expected to have its own *h*_ABL_ value, depending on its diffusivity. According to Pohl *et al*. [11], such "theoretical predictions ...[are]... widely ignored." Moreover, using ion-selective microelectrodes, Pohl and coworkers unequivocally showed that *h*_ABL_ varied with ionic substances at a given level of stirring.

In this Communication, it is hypothesized that the theoretical α = 0.5 was obscured in prior Caco-2 and PAMPA microtitre plate permeability studies [5-9], either because (i) *K* was evaluated without explicit consideration of the *D*_aq_ term from the Levich equation, and/or (ii) the data were not of sufficient sensitivity to reveal the theoretical values. We proceeded to test the hypothesis with PAMPA data by re-arranging the Levich equation into a parametric form. Combining *P*_ABL_ = *D*_aq_ / *h*_ABL_ with Eq. (2) and converting into the logarithmic form:


(3)

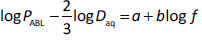



with the theoretical constants a = log (0.201 w^-1/6^) = -0.356 (25 °C) and b = 0.5. We applied Eq. (3) to the (*P*_ABL_, *f*) data of Avdeef *et al.* [9], augmented with additional measurements at 21 and 313 RPM (Table 1), and found a = -0.731 and b = 0.505 (r^2^ = 0.93, SD = 0.09, F = 50, n = 6). The plot of the data used in the re-analysis is shown in Figure 1. The slope factor, 0.505, is so close to the theoretical value that we propose to simply use the theoretical value henceforth. Substituting the new parameters into Eq. (3) and converting the resulting equation to the form of Eq. (2) results in:


(4)

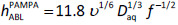



From Eqs. (2) and (4), *h*_ABL_^PAMPA^/*h*_ABL_^Levich^ = 2.4. The geometry of the rotating disk apparatus allows the convective flow to reach closer to the rotating surface (thus diminishing the thickness of the pure diffusion layer) compared to the geometry of magnetically stirred PAMPA wells. The stirring of Caco-2 plates by orbital shakers produces even a greater ratio, *h*_ABL_^Caco-2^/*h*_ABL_^Levich^, indicating less "efficient" stirring [9]. Table 2 shows sample calculations using Eqs. (2) and (4) for three drugs, widely ranging in size.

Pohl *et al.* [11] suggested that if a single reference compound is used to calibrate the geometrical factor, then calculations of subsequent *h*_ABL_ should be according to the diffusivity dependence in the Levich equation:


(5)

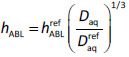



Eq. (5) was experimentally verified with several combinations of ions and buffers by Pohl *et al.* [11], using *pH* and other ion-selective microelectrodes to directly measure the change in concentrations in the aqueous boundary layer adjacent to black lipid membranes.

In conclusion, the stirring frequency exponent of -1/2 in the theoretical Levich expression appears to apply to PAMPA assays, where efficient individual-well magnetic stirring (> 20 RPM) is used. The same may be true for Caco-2 assays, although additional measurements at varied stirring speeds would make this a more confident assertion. If a single molecule is used as a stirring calibrant, then it seems reasonable to use the scaling suggested by Eq. (5) with microtitre plate data. As Table 2 suggests, the error in calculating *h*_ABL_ based on unscaled *h*_ABL_^ref^ can be as high as 30 %. Hence, it is prudent to incorporate Eq. (5) in the calibration procedure. This is especially important to bear in mind for nonionizable molecules since the p*K*_a_^flux^ method cannot be directly applied to them. This is of practical importance in PAMPA and perhaps cellular assays as well.

## Figures and Tables

**Figure 1.**
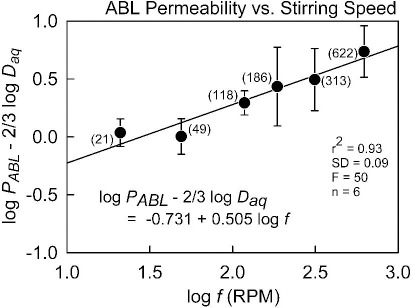
The averaged log *P*_ABL_ - 2/3log*D*_aq_ vs. log *f* (RPM) plot of ionizable molecules, with PAMPA measurements done at six different stirring speeds. The data are from Avdeef et al.[9], augmented with previously unpublished measurements at 21 and 313 RPM. The values in parentheses refer to the RPM values.

**Table 1.** Aqueous boundary layer permeability data

*f* (RPM)	log *P*_ABL_ - ⅔ log *D*_aq_^a^	*SD*	*n* ^b^
21	0.037 ^c^	0.119	15
49	0.004 ^d^	0.154	5
118	0.294 ^d^	0.104	6
186	0.435 ^d^	0.340	51
313	0.495 ^c^	0.268	49
622	0.737 ^d^	0.222	22

^a^ *P*_ABL_ is aqueous boundary layer permeability determined by the p*K*_a_^flux^ method [2,8,9].

^b^ Number of measurements averaged.

^c^ This work.

^d^ Values averaged from ref [9].

**Table 2.** Aqueous boundary layer (ABL) thickness at 300 RPM

COMPOUND	*t* (℃)	*ν* (cm^2^/s) ^a^	*D*_aq_ (cm^2^/s) ^b^	*h*_ABL_^Levich^ (μm) ^c^	*h*_ABL_^PAMPA^ (μm) ^d^
vincristine	25	0.00893	3.49E-06	20	33
	37	0.00697	4.67E-06	21	35
testosterone	25	0.00893	5.58E-06	23	39
	37	0.00697	7.47E-06	25	41
benzoic acid	25	0.00893	8.21E-06	26	44
	37	0.00697	1.10E-05	28	47

^a^ Values of kinematic viscosity, V, were taken from Riddick and Bunger [12].

^b^ Diffusivity, *D*_aq_, calculated by the procedure described elsewhere [8].

^c^ Values of the ABL thicknesses, *h*_ABL_^Levich^, were calculated by Eq. (2).

^d^ *h*_ABL_^PAMPA^ calculated by Eq. (4).
